# Antibiofilm and Enzyme Inhibitory Potentials of Two Annonaceous Food Spices, African Pepper (*Xylopia aethiopica*) and African Nutmeg (*Monodora myristica*)

**DOI:** 10.3390/foods9121768

**Published:** 2020-11-29

**Authors:** Alfred Ngenge Tamfu, Ozgur Ceylan, Selcuk Kucukaydin, Mehmet Ozturk, Mehmet Emin Duru, Rodica Mihaela Dinica

**Affiliations:** 1 Department of Chemical Engineering, School of Chemical Engineering and Mineral Industries, University of Ngaoundere, Ngaoundere 454, Cameroon; 2 Food Quality Control and Analysis Program, Ula Ali Kocman Vocational School, Mugla Sitki Kocman University, 48147 Ula Mugla, Turkey; ozgurceylan@mu.edu.tr; 3 Department of Chemistry Physical and Environment, Faculty of Sciences and Environment, “Dunarea de Jos” University of Galati, 111 Domneasca Street, 800201 Galati, Romania; 4 Department of Medical Services and Techniques, Koycegiz Vocational School of Health Services, Mugla Sitki Kocman University, 48800 Mugla, Turkey; selcukkucukaydin@gmail.com; 5 Department of Chemistry, Faculty of Science, Mugla Sitki Kocman University, 48000 Mugla, Turkey; mehmetozturk@mu.edu.tr (M.O.); eminduru@mu.edu.tr (M.E.D.)

**Keywords:** African food spices, GC-MS (gas chromatography mass spectrometry), antimicrobial, antibiofilm, violacein inhibition, swarming inhibition, swimming inhibition, anticholinesterase, antiurease, antityrosinase

## Abstract

Food pathogens represent an important health threat, and it is relevant to study the effect of foodstuffs such as spices which can inhibit bacterial growth. This study reports the antimicrobial, antibiofilm, and enzyme (Acetylcholinesterase, Butyrylcholinesterase, urease, tyrosinase) inhibitory activities of two medicinal food spices belonging to the Annonaceae family, *Monodora myristica* and *Xylopia aethiopica*. GC-MS (gas chromatography mass spectrometry) analysis of silylated samples of Methanol-Dicloromethane (50:50) extracts of both plants led to the identification of nine compounds in *M. myristica* and seven compounds in *X. aethiopica*. *M. myristica* and *X. aethiopica* had the same minimum inhibitory concentration (MIC) values of 0.625 mg/mL and 2.5 mg/mL on *C. albicans* and *E. coli*, respectively. However, *M. myristica* had better activity than *X. aethiopica* on *Staphylococcus aureus*, while *Pseudomonas aeruginosa* was more susceptible to *X. aethiopica* than *M. myristica*. The lowest MIC value was 0.1325 mg/mL, exhibited by *M. myristica* on *S. aureus*. Both extracts showed good antibiofilm activity. On *S. aureus*, at the same concentration, *M. myristica* had better antibiofilm activity than *X. aethiopica*. On *E. coli* and *Candida albicans*, *X. aethiopica* had better antibiofilm activity than *M. myristica* at the same concentration. *X. aethiopica* showed better violacein inhibition in *Chromobacterium violaceum* CV12472, as its percentage inhibition of violacein varied from 80.5% ± 3.0% at MIC to 5.6 ± 0.2 at MIC/8, as compared to *M. myristica* with 75.1% ± 2.5% at MIC and 15.5% ± 1.1% at MIC/8. The anti-motility activity by swimming and swarming inhibition on *P. aeruginosa* PA01 was low at test concentrations and in both models, *M. myristica* showed higher motility inhibition than *X. aethiopica*. Although in enzyme inhibitory assays all extracts had low inhibitions compared to standards tested at the same concentrations, the results show that these plants can be used to manage food-borne infections.

## 1. Introduction

In every region of the world, selected indigenous plants are used as foods and spices, and investigating their chemical composition and bioactivities has become an interesting field of research. This is because it reveals both the nutritive value and medicinal potential of these food materials used as remedies especially for recalcitrant infectious diseases resulting from food contamination. Spices are defined by Corn et al. (1999) as ingredients usually from vegetables or different dried plant parts like barks, seeds, and leaves added in nutritionally small quantities to food in order to improve its color, taste, or flavor and may also play the role of preservatives that inhibit harmful bacterial growth [1,2]. Most spices are added to food recipes primarily for flavoring, seasoning, and imparting aroma to foods rather than for their nutritional benefits, and for this reason, their phytochemical compositions as well as bioactivities are still under-studied [3]. There is need for the search of locally available nutritional food excipients in Africa and other low income countries which are plagued with poor nutrition [4]. *Xylopia aethiopica* and *Monodora myristica* are two widely consumed food spices in Africa and both belong to the Annonaceae family of plants and have proven to be potent in managing microbial and fungal infections [5]. Annonaceous plants are commonly called custard apples and comprises over 2300 species which possess good biological activities [6].

*X. aethiopica*, also called African pepper, is a highly consumed food spice in Africa and it is used traditionally to manage rheumatism, bronchitis, headache, asthma, stomach-aches, neuralgia, dysenteric conditions, wounds and sores, constipation, epilepsy, fertility, and the ease of childbirth [7]. It has been shown to possess anticancer, antidiabetic, antimalarial, antioxidant, enzyme inhibitory, antimicrobial, and antibacterial properties and also protects against liver and kidney damage [3,7,8,9,10,11,12,13,14].

*Monodora myristica*, also called African nutmeg, bears many fruits. The seeds of this plant are mostly used in dry powdered form which is used as spice in desserts, stews, soups, and cakes. It is also marketed as whole seeds. These plants’ seeds and powders are used in repelling insects, as a stimulant, and as remedy for sores, headache, and stomach disorders. In medicine, the bark is used in treatments of stomachaches, febrile pains, eye diseases, and hemorrhoids [15,16]. This plant possesses antioxidant, antisplasmodic, antiulcer, antimicrobial, cytotoxic, antinociceptive, anti-inflammatory, anticancer, and hepatoprotective activities, and it has been proven in male Wistar rats with induced hypercholesterolemic to modulate lipid peroxidation and bring down cholesterol levels [3,17,18,19,20,21,22,23,24,25].

These plants have been reported to possess antimicrobial activities, with more emphasis on their essential oil and little information about their extracts, meanwhile the plant seeds are consumed in whole. No studies have reported the ability of these plants to inhibit quorum-sensing-mediated effects in bacteria such as biofilm, violacein production, and swarming and swimming motilities. The aim of this work is to evaluate the ability of *M. myristica* and *X. aethiopica* extracts to inhibit biofilm formation, violacein production, and swimming and swarming motilities, as well as acetylcholinesterase, butyrylcholinesterase, urease, and tyrosinase enzymes.

## 2. Materials and Methods

### 2.1. Plant Material and Extraction

The seeds of *Xylopia aethiopica* and *Monodora myristica* were purchased from the Bamenda food market. Both spicy plants were identified by Mr. T. Fulbert, a botanist working at the National Herbarium of Cameroon and compared with existing voucher specimens 28725/SRF Cam. (*X. aethiopica*) and 49544/HNC (*M. myristica*). Of each of the seeds, 200 g were powdered and subjected to maceration extraction. Then, 2 L of dichloromethane/methanol mixture in the ratio 1:1 was used as the solvent for extraction process and the mixture was allowed to stand at room temperature for 48 h with intermittent stirring. After this, the supernatant was carefully decanted and filtered using a Whatman number 1 filter paper. This filtrate was evaporated on a Rotary evaporator to remove the solvent. This process was repeated three times for each sample to yield crude extracts of *X. aethiopica* (27 g) and *M. myristica* (41 g).

### 2.2. GC-FID (Gas Chromatography Flame Ionization Detector) and GC-MS Analyses

Prior to the GC-MS analyses, the samples were silylated using BSTFA [bis (trimethylsilyl)-trifluoroacetamide], according to the method described by Talla and coworkers [26]. GC-FID and GC-MS were performed as described elsewhere [27]. The GC-MS profiles of the extracts were achieved on a gas chromatograph (Hewlett-Packard 5890, Bunker Lake Blvd, Ramsey, MN, USA) with a JEOL MS-600H mass spectrometer (Tokyo, Japan) as detector. Prior to this, GC-FID was performed on a Shimadzu GC-17(Shimadzu Corp., Kyoto, Japan). We used helium (1 mL/min) as the carrier gas at a split ratio of 1:10 in a SPB-5VR capillary column of length 30 m and inner diameter 0.25 mm. The initial temperature of the oven was varied as follows: 60 °C for 3 min, increased at the rate of 5 °C/min to 180 °C, and finally at 7 °C/min to 300 °C final temperature. A ZB-5MSVR column of 30 m length and 0.25 mm inner diameter was used for GC-MS and the same temperature conditions as for GC-FID were applied. Next, 250 °C and 70 eV was applied for the ion source. Mass spectral fingerprints were used for identification on the NIST library and compared with some data reported.

### 2.3. Determination of MIC (Minimal Inhibitory Concentration)

The bacterial and fungal strains *Staphylococcus aureus* (ATCC 25923), *Escherichia coli* (ATCC 25922), *Candida albicans* (ATCC 10239), *Chromobacterium violaceum* (CV12472), and *Pseudomonas aeroginosa* (PA01) were used.

The broth dilution method, as described by the CLSI (Clinical and Laboratory Standards Institute, 2006), was applied to determine the MIC values [28]. The lowest concentration of extract at which no bacterial growth was visible was considered as the MIC. Mueller-Hinton Broth (MHB) was used as the medium and the bacterial concentration used had a density of 5 × 10^5^ colony-forming units (CFU)/mL. Into 96-well microtiter plates, containing extracts at concentrations (10, 5, 2.5, 1.25, 0.625, 0.312 mg/mL), 100 μL of microbial cell solutions were inoculated and incubated for 24 h at 37 °C, after which the MICs were determined and recorded.

### 2.4. Assay of Inhibition of Bacterial Biofilm Formation by Extracts

The ability of the extracts to prevent biofilm formation by bacteria (*S. aureus, E. coli* and *C. albicans*) was evaluated at concentrations of 1, 1/2, 1/4, 1/8, and 1/16 (MIC) using the microplate antibiofilm method [29]. Tryptose-Soy Broth (TSB) containing 0.25% glucose were filled into wells with or without extracts, and 200 μL of 1% overnight bacterial cell cultures (5 × 10^5^ CFU/mL) were added and incubated at 37 °C for 48 h. The negative control wells contained only TSB and bacterial cells. The planktonic bacteria were washed after incubation, and the remaining bacteria were stained with 0.1% crystal violet solution and allowed to sit for 10 min. The dye was washed out carefully with distilled water, after which 200 μL of ethanol or 33% glacial acetic were filled into the wells of the microplates. Next, 125 μL of the resulting solution were transferred using a pipette into sterile tubes and the total volume was made up to 1 mL by adding distilled water. The optical density of each tube was read at 550 nm, and the equation below was used to calculate the percentage inhibition of the biofilm formation. Each experiment was done three times.

$$\mathrm{Biofilm}\;\mathrm{inhibition}\;\left(\%\right)\;=\;\frac{OD\;550\;control-OD\;550\;sample\;}{OD\;550\;control}\times 100$$

### 2.5. Violacein Pigment Inhibition Assay

This assay measures the qualitative quorum-sensing potential of the extracts using *Chromobacterium violaceum* ATCC 12472 strain [30]. *C. violaceum* CV12472 was grown overnight and 10 µL of it were put into microtiter sterile plates filled with 200 µL of LB broth. Sub-MIC concentrations of extracts were added and then incubated at 30 °C for 24 h. Control plates contained only LB broth and *C. violaceum* ATCC 12472. The decrease in the production of violacein pigment was measured by taking the absorbance at 585 nm. The violacein inhibition percentage of the extracts were calculated as follows:

$$\mathrm{Violacein}\;\mathrm{pigment}\;\mathrm{inhibition}\;\left(\%\right)\;=\;\frac{OD\;585\;control-OD\;585\;sample}{OD\;585\;control}\times \;100\;$$

### 2.6. Swimming and Swarming Motility Inhibition on Pseudomonas Aeruginosa PA01

The ability of the extracts to inhibit swarming motility in *P. aeruginosa* PA01 was performed as described elsewhere [30]. Briefly, swarming plates consisting of, 0.5% NaCl, 1% peptone, 0.5% agar, and 0.5% D-glucose together with extracts at three concentrations of 50, 75, and 100 µg/mL were prepared. *P. aeruginosa* PAO1 was grown overnight and 5 µL of it were point-inoculated at the center, and the swarm plates were wrapped with paraffin and inoculated in an upright position for 18 h. The plates not containing extracts were used as controls. The swarming movement was measured from the swarm diameter fronts.

The swimming plates consisted of 1.5% agar, 1% peptone, 0.5% NaCl, and 0.5% D-glucose together with the extracts at 50, 75, and 100 μg/mL concentrations. The same bacteria *P. aeruginosa* PAO1 was inoculated as in the swarming model. The inoculation was done for 18 h and control plates did not contain extracts. Swim zone diameter for samples and controls were used in calculating the percentage inhibition of swimming motility.

### 2.7. Cholinesterase Inhibition Assay

Ellman’s Method was used to evaluate the acetylcholinesterase (AChE) and butyrylcholinesterase (BChE) inhibition potential of extracts, and slight modifications were made [31]. Next, 96-well microplates were used and acetylthiocholine iodide (0.71 mM) was the substrate in AChE assay, while butyrylthiocholine chloride (0.2 mM) was the substrate for the BChE assay. In a 96-well plate containing 150 μL sodium phosphate buffer 100 mM (pH = 8), 10 μL of the sample were mixed with 20 μL of enzymes AChE or BChE. The mixture was incubated at 25 °C for 15 min, after which 10 μL of substrates and 10 μL of Ellman’s Reagent (DTNB 0.5 mM) were added and the volume made up to 200 μL. The absorbance was measured at 412 nm for 10 min. The percentage inhibition of AChE or BChE was determined control using the formula:

(E − S)/E × 100
(1)

where:

E: activity of enzyme with control.

S: activity of enzyme with sample.

The experiments were repeated three times. Galantamine was used as the standard.

### 2.8. Tyrosinase Inhibition Assay

A spectrophotometrical method was used to evaluate the anti-tyrosinase activity of extracts in which tyrosinase enzyme from mushrooms was used following a method described elsewhere with a slight modification [32]. The substrate used in this assay was L-Dopa, while kojic acid was used as the standard inhibitor of tyrosinase. The percent inhibition of the enzyme (Inhibition %) by the extracts was calculated at each sample concentration (µg/mL) in a similar manner as in the AChE and BChE assay.

### 2.9. Urease Inhibition Assay

The indophenol method, in which the production of ammonia is measured, was used to determine the potential of extracts to inhibit urease [33]. A mixture of 25 μL of a Jack bean source urease enzyme, phosphate buffer 100 mM (pH 8.2), and 50 μL of urea 100 mM was prepared and after the adding of the samples (10 μL, 1 mM), it was incubated for 15 min at 30 °C. Subsequently, 45 μL of phenol reagent 1% (*w*/*v*) and 70 μL of 0.005% (*w*/*v*) alkali reagent were both added into each well and the mixture further incubated for 50 min. The standard used was Thiourea. The absorbances were recorded at 630 nm and the % inhibitions were calculated.

### 2.10. Statistical Analysis

Each activity was done in triplicate. The results were recorded as the means ± standard error of the mean. Fisher’s test was used to determine the significant differences between means; *p* < 0.05 were regarded as significant.

## 3. Results

### 3.1. GC-MS Chemical Composition

**Figure 1:**
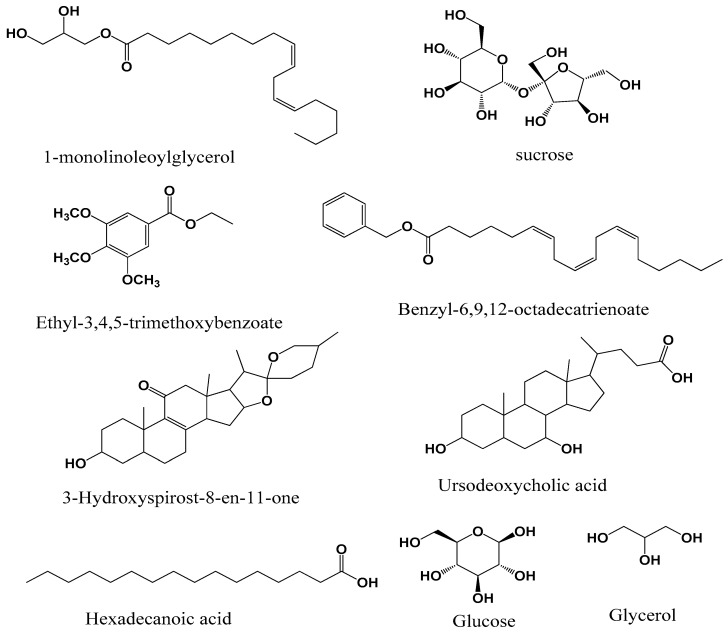
Chemical compounds identified in *M. myristica* seed extract by gas chromatography mass spectrometry (GC-MS).

**Figure 2:**
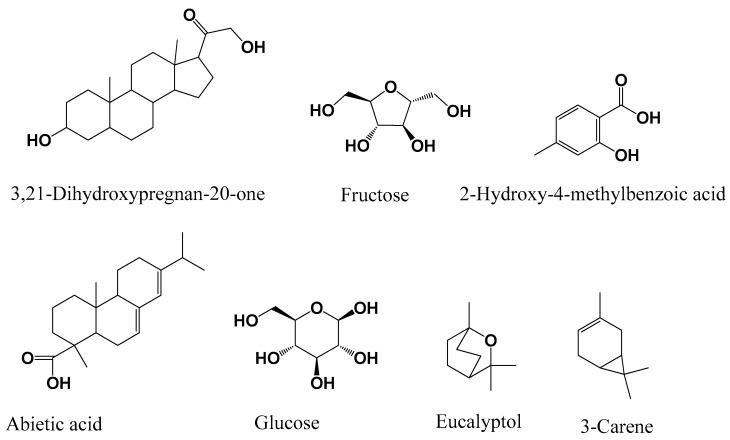
Chemical compounds identified in *X. aethiopica* seed extract by GC-MS.

Nine compounds (Figure 1), including 1-monolinoleoylglycerol, 6,9,12-octadecatrienoic acid benzyl ester, 3-hydroxyspirost-8-en-11-one, ethyl-3,4,5-trimethoxybenzoate, palmitic (hexadecanoic) acid, ursodeoxycholic acid and sugars such as glycerol, glucose, and sucrose were identified in *M. myristica*. On the other hand, seven compounds (Figure 2), were identified in *X. aethiopica*, these were 3-carene, eucalyptol, 2-hydroxy-4-methylbenzoic acid, abietic acid, 3,21-dihydroxypregnan-4-one alongside two sugars, fructose, and glucose.

### 3.2. Minimal Inhibitory Concentrations

The minimal inhibitory concentrations (MICs) of both extracts are reported in Table 1. Both plants *X. aethiopica* and *M. myristica* had the same MIC values of 0.625 mg/mL and 2.5 mg/mL on *C. albicans* and *E. coli*, respectively. However, *M. myristica* had better activity than *X. aethiopica* on *S. aureus,* while *P. aeruginosa* was more susceptible to *X. aethiopica* than *M. myristica*. The lowest MIC value was 0.1325 mg/mL, exhibited by *M. myristica* on *S. aureus*.

### 3.3. Percentage Biofilm Inhibition

Prior to antibiofilm assay, the MIC values were determined and the biofilm inhibition potential of both extracts determined at MIC and sub-MIC concentrations and reported in Table 2. Both extracts had good antibiofilm activity on *S. aureus*. On *S. aureus*, the biofilm inhibition of *X. aethiopica* varied from 45.3% ± 1.5% at MIC to 5.2% ± 1.0% at MIC/8 while that of *M. myristica* varied from 52.6% ± 3.3% at MIC to 11.3% ± 1.5% at MIC/8. On *S. aureus*, at the same concentration, *M. myristica* had better antibiofilm activity than *X. aethiopica*. On *E. coli* and *C. albicans*, *X. aethiopica* had better antibiofilm than *M. myristica* at the same concentration (*p* < 0.01).

### 3.4. Violacein and Motility (Swimming and Swarming) Inhibition Percentages

The percentage inhibition of violacein pigment synthesis by *C. violaceum* CV12472 was evaluated at MIC and sub-MIC concentrations and reported in Table 3. *X. aethiopica* inhibited violacein production better than *M. myristica*, as it percentage inhibition of violacein varied from 80.5% ± 3.0% at MIC to 5.6 ± 0.2 at MIC/8 as compared to *M. myristica* with 75.1% ± 2.5% at MIC and 15.5% ± 1.1% at MIC/4. At MIC/16, no inhibition was observed.

The swimming and swarming inhibition assay was carried on *P. aeruginosa* PA01 at 100 µg/mL, 75 µg/mL, and 50 µg/mL, and the results are given in Table 3. The anti-motility activity of these plant extracts were low at test concentrations. In both models, *M. myristica* showed higher motility inhibition than *X. aethiopica* (*p* < 0.05).

### 3.5. Enzyme Inhibitory Activities

The capacity of *X. aethiopica* and *M. myristica* to inhibit some selected enzymes acetylcholinesterase, butyrylcholinesterase, tyrosinase, and urease are evaluated and reported in Table 4. On AChE, the activities of *X. aethiopica* (7.80% ± 0.36%) and *M. myristica* (9.65% ± 0.11%) were almost the same but far lower than that of the standard galantamine (80.76% ± 0.52%). This was the same effect for BChE on which percentage inhibitions of *X. aethiopica* and *M. myristica* were 15.48 ± 0.57 and 13.64 ± 0.88 respectively against 74.43% ± 0.30% for galantamine.

*X. aethiopica* (30.45% ± 0.84%) had better inhibition on tyrosinase than *M. myristica* (23.44% ± 1.27%) while on urease, *M. myristica* (15.18% ± 0.86%) was more active than *X. aethiopica* (5.69% ± 0.33%).

## 4. Discussion

The chemical composition of both plant extracts were effected using GC-MS. The GC-MS was done after the samples were silylated to enable the detection of a large number of compounds including polar ones. Silylation helps to reduce polarity of compounds contained in the extract while equally rendering them more volatile such that they become detectable on GC-MS. These enabled the identification of major compounds in both extracts. These two plants investigated in this study have evidently been proven to possess antimicrobial activity and have been used extensively to manage infectious diseases [34], but much of this has been attributed to its essential oils [5] with little attention on the extracts. In this study, the extracts have also demonstrated appreciable antimicrobial activity, and this is advantageous, since the plants are consumed principally in crude form. Antimicrobial activity was displayed by both plant extracts and these results corroborate with some findings in which these plant extracts showed antimicrobial activity against some pathogens [35]. However, microbial resistance arises due to quorum-sensing-mediated traits of pathogens which determines the severity of infections as well. The assays that involved quorum-sensing-mediated processes in bacteria are usually focused biofilm formation, violacein pigment production, and swimming and swarming motilities. Prior to these assays, MIC values are determined and they are performed at MIC and sub-MIC concentrations. Thus, working at sub-MIC concentrations, the hypothesis of bactericidal effect of extracts that occurs at high concentrations is eliminated, giving way for QS investigation.

In order to protect themselves during adverse conditions such as immunological defense systems of host, antibiotics, and starvation, many fungi and bacteria cells constitute self-organized and three-dimensional communities in which they will live. These constituted and protected communities are called biofilms and they contribute to the severity of chronic infections as well as the persistence of resistance to drugs and antibiotics [6,36]. Therefore, most antimicrobial agents treat symptoms of planktonic bacteria, and disease will resurface, due to the bacteria which were within biofilms and which will break out when the threat from the antibiotic is over. The potential of these plant extracts to inhibit biofilm formation by test bacteria is a very desirable effect. This can subsequently eliminate bacteria resistance or reduce the severity of microbial infections.

Violacein (violet color) production is one of the quorum-sensing-mediated traits of pathogenic bacteria *C. violaceum* that has received maximum attention and has the advantage that it is easily measurable and quantifiable. Violacein is a biomolecule that has antimicrobial activity against other microorganisms and also improves the *C. violaceum’s* ability to cope with environmental stress [37,38]. *C. violaceum* has been highly applied in evaluating the antiinfective potential of many natural and synthetic products, and those with violacein inhibition find application potential alternatives to conventional bactericidal antibiotics.

The bacterial communities that are formed on surfaces constitute a serious health threat and potent contamination. The colonization of various surfaces by bacteria through bacterial motility can be reduced or limited by the use of natural substances and compounds which need to be identified and evaluated for their applications to impede bacterial colonizations [39]. Before forming biofilms, bacteria move by swimming and swarming and colonize surfaces, and this step is believed to be an initial step for the formation of the biofilm in flagellated *P. aeruginosa* PA01. This bacterium can use its flagella to swim towards surfaces followed by swarming and twitching motilities, which enables it to colonize surfaces and establish biofilm communities.

Cholinesterase inhibitors are a group of medicines that block the normal breakdown of acetylcholine. Deterioration of the central nervous system and Alzheimer’s disease (AD) result from cholinergic deficiency and are associated with dementia. As a remedy to this situation, various cholinesterase inhibitors such as natural compounds and extracts and synthetic analogues and their hybrids are being employed [40]. Natural medicines are gaining ground because of their low toxicities and few side effects compared to synthetic ones. The enzyme-mediated browning reaction in harvested fruits is usually initiated by the copper-containing oxidase enzyme called tyrosinase. It is an enzyme with multiple functions and is responsible for the undesirable over-pigmentation of human skin in a process that is similar to that which takes place during the browning of fruits. The first two steps in mammalian melanogenesis are catalyzed by this enzyme, and hence the search for potent tyrosinase inhibitors, especially those that can be used safely in cosmetics and foods, is an attraction for researchers [41]. Many bacteria produce the nickel-dependent urease (ureolytic bacteria) enzyme, which is capable of hydrolyzing urea to produce ammonia with the emission of carbon dioxide as well. The emission of ammonia from agriculture is usually associated with these bacteria, and it also causes a number of infectious diseases [42]. Inhibiting urease is beneficial to human health and some notorious bacteria depend on urease-mediated processes for survival. Although in the enzyme inhibitory assays no extract had an inhibition close to that of the standards, these results are moderate because the extracts were tested at the same concentrations as the pure standard compounds.

Many of the compounds identified in these plants have previously been described in some medicinal plant extracts. 1-monolinoleoylglycerol, which is found in *M. myristica* has been described in the extract of *Datura stramonium* and *Salvadora persica* and the extracts of these plants have shown antimicrobial and anticancer activities [43,44]. Certain sugars were detected in both *M. myristica* and *X. aethiopica* plant extracts notably sucrose, glucose, glycerol, and fructose. These sugars were shown to possess antibacterial activities [45,46]. Ethyl-3,4,5-trimethoxybenzoate detected in *M. myristica* and other derivatives of 3,4,5-trimethoxybenzoic acid have been identified in medicinal plant *Mitracarpus scaber* and have demonstrated antimicrobial properties [47], and *Anarcadium occidentale* (cashew gum) also has been synthesized and evaluated for antioxidant and enzyme inhibition [48]. 6,9,12-octadecatrienoic acid phenylmethyl ester was found in the extract of *M. myristica*, and this compound was detected in *Croton bonplandianum* extract which possesses anti-inflammatory and enzyme inhibitory potentials [49]. 3-hydroxyspirost-8-ene-11-one, which was detected in *M. myristica*, has been previously described in *Artemisia annua*, and this plant extract studied showed antioxidant, antimicrobial, and anti-inflammatory activities [50]. Ursodeoxycholic acid was identified in *M. myristica,* and this compound has been synthesized from a natural source [51]. Hexadecanoic acid contained in *M. myristica* has been described in many plants and this compound has good medicinal properties including anti-inflammatory, antioxidant, and enzyme inhibitory activities including anticholinesterase activity [52,53,54]. The compound 2-Hydroxy-4-methylbenzoic acid present in *X. aethiopica* has been detected previously in *Hemidesmus indicus* and mangrove fungus and has displayed anti-inflammatory, antipyretic, and antioxidant activities [55,56]. In the extract of *X. aethiopica*, abietic acid was identified, and this compound has been detected in *Isodon wightii*, which displayed antibacterial, antioxidant, and anticholinesterase activities [57]. A major component of eucalyptus called eucalyptol or 1,8-cineole identified in *X, aethiopica* extract has been detected in many species of eucalyptus, whose extracts possess antimicrobial and antiviral activities [58,59]. The volatile compound 3-carene was detected in the extract of *X. aethiopica* and has been described as an antimicrobial compound [60]. From these analyses of previous studies, it can be understood that the antimicrobial, antibiofilm, and enzyme inhibitory activities of these extracts of *M. myristica* and *X. aethiopica* can be attributed to these chemical compounds that they contain.

## 5. Conclusions

The investigation of bioactivities of food extracts and food-derived substances is attracting much attention because of their considerable and known safety. These food substances are exploited for their medicinal properties besides their nutritive values especially to combat food-borne infections resulting from various food pathogens. The emergence and spread of drug-resistant strains nowadays threatens the use of conventional antibiotics in order to treat bacterial infections and diseases. New strategies such as biofilm inhibition and quorum-sensing disruption are therefore necessary to overcome persistent infections especially those that involve resistant biofilm formation by the pathogens. Both food spices *X. aethiopica* and *M. myristica* have shown biofilm inhibitory potentials and also violacein inhibition and swimming/swarming motilities inhibitions. These traits are quorum-sensing-mediated processes which help to increase the resistance of bacteria and virulence of their infections. Both extracts displayed low to moderate anticholinesterase activities and tyrosinase and urease inhibitory potentials, which is interesting for these natural food spices. As an added value to the antimicrobial properties of these two natural food spices, they can be desirable as enzyme inhibitors which may present no or milder side effects than conventional synthetic drugs used for this purpose. The bioactivities of these extracts opens a space for the search of the bioactive molecules contained in them.

## Figures and Tables

**Table 1:** Antimicrobial activities of *X. aethiopica* and *M. myristica* (minimum inhibitory concentration (MIC) values in mg/mL).

Microorganisms	*Extracts*	
	*X. aethiopica*	*M. myristica*
*C. albicans* ATCC 10239	0.625	0.625
*S. aureus* ATCC 25923	0.625	0.3125
*E. coli* ATCC 25922	2.5	2.5
*C. violaceum* CV1247*2*	2.5	1.25
*P. aeruginosa* PA01	0.625	1.25

**Table 2:** Effects of different concentrations of *X. aethiopica* and *M. myristica* extracts on tested bacteria biofilm formations.

Strains	Conc.	*X. aethiopica*	*M. myristica*
		% of Biofilm Inhibition	
*S. aureus* ATCC 25923	MIC	45.3 ± 1.5 *	52.6 ± 3.3
	MIC/2	30.0 ± 3.4	39.0 ± 1.4
	MIC/4	16.7 ± 0.4	23.8 ± 0.6
	MIC/8	5.2 ± 1.0	11.3 ± 1.5
	MIC/16	-	-
*E. coli* ATCC 25922	MIC	21.0 ± 2.5	18.6 ± 0.5
	MIC/2	10.5 ± 0.5	8.3 ± 2.0
	MIC/4	-	-
	MIC/8	-	-
	MIC/16	-	-
*C. albicans* ATCC 10239	MIC	31.7 ± 1.5	29.6 ± 3.3
	MIC/2	22.4 ± 1.3	15.3 ± 0.1
	MIC/4	09.2 ± 0.5	8.9 ± 2.0
	MIC/8	-	-
	MIC/16	-	-

*: Data are the mean of three replicates ± SD. Statistically different (*p* < 0.01).

**Table 3:** Effects of *X. aethiopica* and *M. myristica* extracts at different concentrations on qualitative violacein inhibition and swarming/swimming motility.

	*X. aethiopica*		*M. myristica*	
Conc.	% of Violacein Inhibition (on *C. violaceum* CV12472)			
MIC	80.5 ± 3.0 *		75.1 ± 2.5	
MIC/2	37.3 ± 0.7		35.8 ± 0.6	
MIC/4	19.4 ± 1.0		15.5 ± 1.1	
MIC/8	5.6 ± 0.2		-	
MIC/16	-		-	
% motility inhibition (on *P. aeruginosa* PA01)				
Conc. (μg/mL)	Swimming inh.	Swarming inh.	Swimming inh.	Swarming inh.
100	25.1 ± 0.5	28.9 ± 2.0	29.2 ± 0.0	31.7 ± 1.2
75	13.6 ± 0.5	12.0 ± 0.5	16.5 ± 0.8	22.3 ± 1.0
50	-	-	-	11.6 ± 0.5

*: Data are the mean of three replicates ± SD. Statistically different (*p* < 0.05).

**Table 4:** Anticholinesterase, antityrosinase, and antiurease activities (%inh.) of test samples at 100 μg/mL.

Sample	AChE	BChE	Tyrosinase	Urease
		% Inhibition		
*X. aethiopica*	7.80 ± 0.36	15.48 ± 0.57	30.45 ± 0.84	5.69 ± 0.33
*M. myristica*	9.65 ± 0.11	13.64 ± 0.88	23.44 ± 1.27	15.18 ± 0.86
Galantamine	80.76 ± 0.52	74.43 ± 0.30	NT	NT
Kojic acid	NT	NT	NT	67.58 ± 0.23
Thiourea	NT	NT	75.17 ± 0.18	NT

NT: Not tested. Statistically different (*p* < 0.01).
